# Supplementary material for: Zinc deficiency and associated factors among pregnant women’s attending antenatal clinics in public health facilities of Konso Zone, Southern Ethiopia
Source: PLoS One. 2022 Jul 7;17(7):e0270971. doi: 10.1371/journal.pone.0270971 (PMC9262205; doi:10.1371/journal.pone.0270971)
Supplement: Attachment — Submitted filename: Response letter.docx [file pone.0270971.s002.docx]

**Response letter**

**#1 Comment:** Is the manuscript technically sounds, and do the data support the conclusions?

The manuscript must describe a technically sound piece of scientific research with data that supports the conclusions. Experiments must have been conducted rigorously, with appropriate controls, replication, and sample sizes. The conclusions must be drawn appropriately based on the data presented.

**Reviewer response to question #1:** The manuscript deals with the important issue of zinc deficiency in pregnant women. The methodology utilized to calculate prevalence ideally should be based on a random sample of the study population. In 2015, an Ethiopia National Micronutrient Survey was implemented with serum samples from over 3,300 subjects, with a 75% (it should be corrected as 72% from the result) (overall prevalence of zinc deficiency). A further systematic review of Ethiopian studies on zinc deficiency was published in 2019, reporting a 60% pooled prevalence of zinc deficiency in pregnant women. Neither of these references are cited in the manuscript. The authors should discuss more thoroughly the possible reasons why they found only 30% prevalence of zinc deficiency in their sample of pregnant women.

**Response** –ok, thanks for your concern, we incorporate the pooled prevalence of Zink deficiency among pregnant women in Ethiopia which is indicated in the introduction and discussion section. We to explain the difference in the prevalence of our study and the two studies. Thank you again for your concern. For study done in 2015, an Ethiopia National Micronutrient Survey was implemented with serum samples from over 3,300 subjects, with a 72% prevalence is the overall prevalence of reproductive, under five children and adults at national level estimate. This result is not comparable with pregnancy women because there is difference in study population.

**Comments:** In this manuscript, there are several methodological issues that should be addressed by the authors.

(1) **Sampling of study sites**. Institution-based sampling could have introduced bias, since a representative random sample of the population was not used. The sample was selected from among women attending prenatal care at one hospital or one of three health centers, which were randomly selected from a group of one hospital and nine health centers. The authors should state what proportion of the total population of pregnant women attended formal prenatal care services. They should clearly describe what group of pregnancy women is represented by the study, and what, if any, possible biases were introduced by sampling from an institutional population.

**Response** – the sample size of study participants were selected based on systematically selected pregnant from 3 health center and one district hospital. Each study participant pregnant mother weekly anti natal follow up were determined from anti natal log registration book. Then proportional to population based allocation approach was implemented for each health facility.

This clearly narrated in sample size determination and procedures section.

(2)**Sampling of study subjects.** The methods for sampling pregnant women within study sites is not described; this should be discussed in detail in the manuscript. The period of time during pregnancy during which blood samples are taken is an important factor for blood assays; this should be described in the manuscript. The authors did take into account timing during pregnancy by assigning different cut-off points of zinc deficiency by first trimester versus second or third trimester of pregnancy. A bibliographic reference needs to be cited to support the cut-off levels chosen. The variation in timing of taking blood samples and interviewing subjects should be discussed in the manuscript, possibly in the limitations section.

**Response –**Ok, thanks, Serum zinc concentration was defined as a serum zinc level of less than 56 μg/dl during the first trimester, and a cut of point less than 50 μg/dl during the second or third trimester and we put it under operational definition in manuscript with bibliographic reference cited. **Regarding –**timing of blood sample collection from the pregnant mothers were collected immediately after interviewing subjects. This helps to keep the blood sample safety. This is narrated under Blood sample collection**.**

(2) **Sample size**. The sample size appears to have been calculated correctly to estimate prevalence. The number of study subjects selected at each site was proportional to the size of the health facility. Thus, it is possible that there was oversampling from the hospital. The authors should discuss the similarity or differences between the characteristics of pregnant women who attended prenatal care in a hospital versus a health center.

**Response** –thanks for your valuable comments- there is no oversampling from the district hospital because the number of study participants were allocated for each health facility proportionally based their monthly follow of pregnant mothers as expanded in the manuscript under sample size determination and procedures section. Regarding similarity or differences between the characteristics of pregnant women who attended prenatal care in a hospital versus a health center. There is no basic difference in terms of ethnicity pregnant women as majority of the study participants were from Konso and Derashe ethnic group with similar age range of 15-49 years in all health facilities. There is no major socio demographic variation of pregnant mother who attended prenatal at health centers and district hospital.

(3) I am unable to comment on the laboratory procedures for the zinc assay.

**Response**-ok thanks we clearly described the standard analytical procedure for zinc level determination. This is clearly narrated in the manuscript under **Zinc level determination from serum sample in method section.**

(4) Co-variates. The important independent variables on nutrition knowledge should be more well-defined in the manuscript, such as “getting nutritional education”, “knowing about balance diet,” knowing about food sources of various types of nutrients, and “meal frequency,” and “dietary diversity.” For example, if a standard 24-hour recall on dietary intake was conducted to calculate the WHO indicator on “dietary diversity,” it should be reported with the appropriate reference cited. Other independent variables such as “wealth index” and “financial freedom” also should be described as to how they were calculated.

**Response-** Ok - nutrition knowledge – variable was measured by using women nutritional knowledge assessment tool which categorized as having good and poor nutritional knowledge. This issue was addressed clearly under operational definition.

Dietary diversity- the dietary diversity score of women was measured based on Minimum Dietary Diversity–Women defined as a dichotomous indicator as low who consumed less than (<5) food items and higher if it is greater than (≥5) out of ten defined food groups during 24 hours. This definition is clearly narrated under operational definition

Well index –was determined by conducting principal component analysis and properly described under manuscript in data analysis section. It was briefly explained under data analysis

Financial freedom- of pregnant mother was measured by using tools that area used in similar setting to determine pregnant mothers financial access to purchase quality food items form the local market without their husband influence which was clearly explain under operational definition with supportive bibliographic reference.

(4) The personal interviews of study subjects in this research should have included a 24-hour dietary recall, which would have identified any consumption of zinc-rich foods. Also, the interview questionnaire of pregnant women should have inquired about specific knowledge of what foods are rich in zinc. If this was done, it should be reported in the manuscript.

**Response** – Ok thanks for valuable comment- During interviews 24 hr. dietary recall methods by using women dietary diversity assessment tools. This briefly explained under data collection part of data collection.

**(5) Conclusions**. The authors need to state the conclusions more precisely and should avoid a tendency for phrases that suggest causality, given that this observational cross-sectional study is only able to identify “associations” between independent variables and the outcome variable of zinc deficiency.

**Response:** Thank you for your scholarly comments -the conclusion of the result further amended based on the comment given and highlighted in the revised manuscript.

(1) Data analysis methods need to be described more clearly. The acronym COR is not spelled out.
**Response –ok**, thanks again for this issue you raised- we explained briefly all data analysis performed during result writing in revised manuscript data analysis section.

COR: Crude odds ratio

(2) The format of presentation is not clear on Table 3 provided. The acronym AOR should be spelled out. The co-variates with an association of p<0.25 which were used for adjustment in the logistic regression should be listed in the text or at the bottom of the table. The final best-fitting logistic model should be clearly shown.

**Response** –ok, we list co-varieties were listed in text manuscript and significant variables were clearly shown in double ** in the association table.

AOR: Adjusted odds ratio under - **Abbreviations section of the manuscript**

(3). Discussion of the findings should be reviewed to align with an understanding of the statistical methods used, such as the statement: “The current study showed that participants whose age was advanced, 35-49 years [AOR 2.59 (1.15, 5.85)] were 2.59 times more likely develop Zink deficiency as compared with those whose age was below 35 years.” This statement should be modified to state that the AOR of 2.59 of the older age group is in comparison to the youngest age group which was used as the reference group.

**Response** –we amend this critical comment under the discussion section by supporting with the existing evidence.

#3. Have the authors made all data underlying the findings in their manuscript fully available?
The PLOS Data policy requires authors to make all data underlying the findings described in their manuscript fully available without restriction, with rare exception (please refer to the Data Availability Statement in the manuscript PDF file). The data should be provided as part of the manuscript or its supporting information, or deposited to a public repository. For example, in addition to summary statistics, the data points behind means, medians and variance measures should be available. If there are restrictions on publicly sharing data—e.g. participant privacy or use of data from a third party—those must be specified.

Response –ok, we have primary data set on SPSSS format as needed we can upload

#4. Any typographical or grammatical errors should be corrected at revision, so please note any specific errors here. The manuscript needs a significant review and correction of English grammar, spelling, and punctuation. There is a repeat of one sentence with the phrase “… contributes 47% of child deaths."

**Response:** we rigorously edited the manuscript grammar, spelling, and punctuation in all section and uploaded as revised manuscript.
